# 2-{[2-(Pyridin-4-yl)-1*H*-benzimidazol-1-yl]meth­yl}phenol

**DOI:** 10.1107/S1600536813009458

**Published:** 2013-04-13

**Authors:** Mohammed A. S. Omer, Jia-cheng Liu, Chao-hu Xiao

**Affiliations:** a College of Chemistry and Chemical Engineering, Northwest Normal University, Lanzhou 730070, People’s Republic of China; b Department of Chemistry, Faculty of Education, University of Khartoum, Sudan

## Abstract

In the title compound, C_19_H_15_N_3_O, the benzimidazole ring system makes dihedral angles of 44.36 (7) and 75.67 (7)° with the pyridine and benzene rings, respectively. In the crystal, phenolic O—H⋯N hydrogen bonds to benzimidazole N-atom acceptors give rise to a chain extending along [011].

## Related literature

For applications of benzimidazole derivatives as ligands, see: Janiak (2000 ▶); Kühler *et al.* (2002 ▶); Li *et al.* (2007 ▶); Carcanague *et al.* (2002 ▶); Yang *et al.* (2006 ▶). For the synthesis of the title compound, see: Fellah *et al.* (2010 ▶). For the structure of a similar compound, see: Kitazume & Ishikawa (1974 ▶).
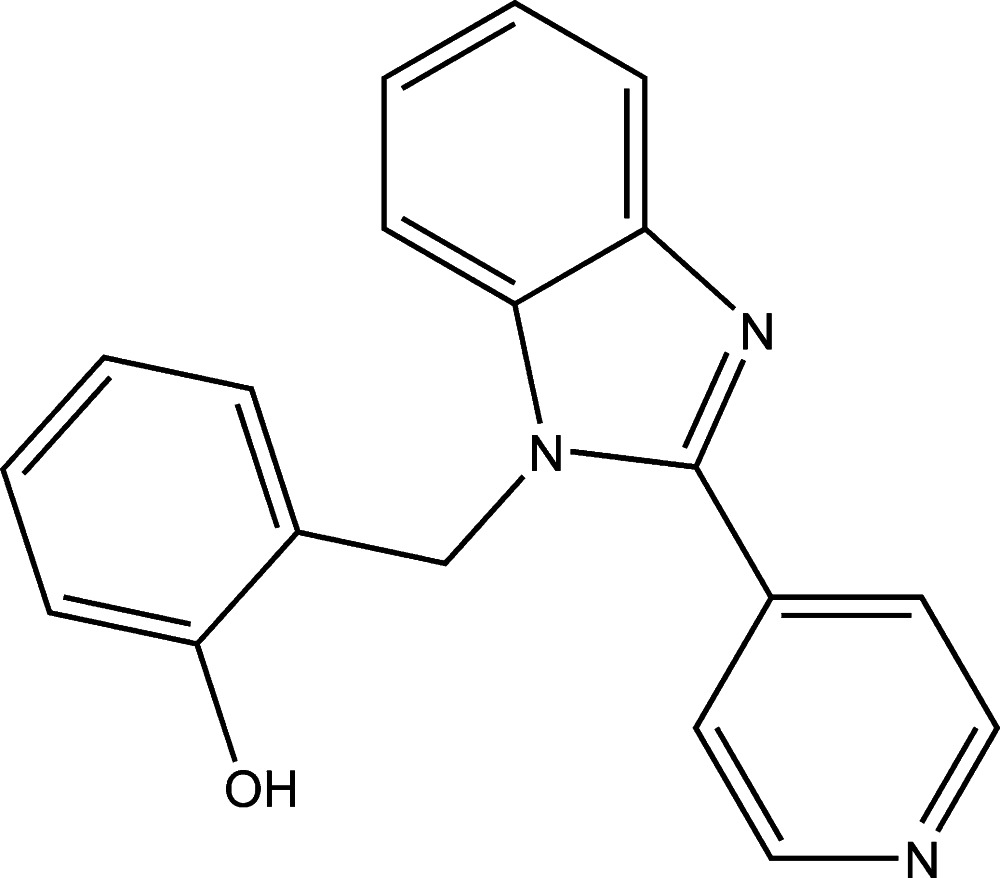


## Experimental

### 

#### Crystal data

C_19_H_15_N_3_O
*M* *_r_* = 301.34
Monoclinic, 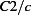

*a* = 16.1886 (7) Å
*b* = 8.4863 (4) Å
*c* = 21.4316 (10) Å
β = 93.756 (4)°
*V* = 2938.0 (2) Å^3^
*Z* = 8
Mo *K*α radiation
μ = 0.09 mm^−1^
*T* = 291 K
0.36 × 0.28 × 0.25 mm

#### Data collection

Agilent SuperNova CCD diffractometer
Absorption correction: multi-scan (*CrysAlis PRO*; Agilent, 2012 ▶) *T*_min_ = 0.982, *T*_max_ = 1.000
6104 measured reflections
3010 independent reflections
2398 reflections with *I* > 2σ(*I*)
*R*_int_ = 0.026

#### Refinement

*R*[*F* ^2^ > 2σ(*F* ^2^)] = 0.047
*wR*(*F* ^2^) = 0.119
*S* = 1.06
3010 reflections
209 parameters
H-atom parameters constrained
Δρ_max_ = 0.14 e Å^−3^
Δρ_min_ = −0.19 e Å^−3^

### 

Data collection: *CrysAlis PRO* (Agilent, 2012 ▶); cell refinement: *CrysAlis PRO*; data reduction: *CrysAlis PRO*; program(s) used to solve structure: *SUPERFLIP* (Palatinus & Chapuis, 2007 ▶); program(s) used to refine structure: *SHELXL97* (Sheldrick, 2008 ▶); molecular graphics: *OLEX2* (Dolomanov *et al.*, 2009 ▶); software used to prepare material for publication: *OLEX2*.

## Supplementary Material

Crystal structure: contains datablock(s) globalk, I. DOI: 10.1107/S1600536813009458/zs2254sup1.cif

Structure factors: contains datablock(s) I. DOI: 10.1107/S1600536813009458/zs2254Isup2.hkl

Supplementary material file. DOI: 10.1107/S1600536813009458/zs2254Isup3.cml

Additional supplementary materials:  crystallographic information; 3D view; checkCIF report

## Figures and Tables

**Table 1:** Hydrogen-bond geometry (Å, °)

*D*—H⋯*A*	*D*—H	H⋯*A*	*D*⋯*A*	*D*—H⋯*A*
O1—H1⋯N2^i^	0.82	1.95	2.7659 (18)	177

Symmetry code: (i) 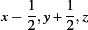.

**Fig. 1.**
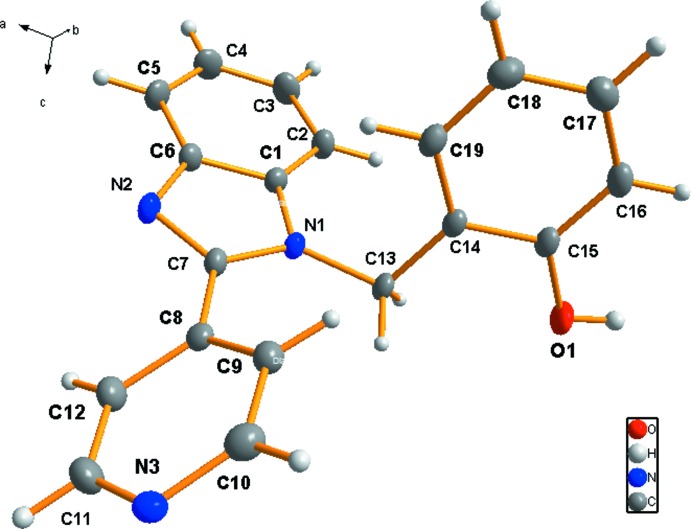
Molecular conformation and atom numbering scheme for the title compound, with displacement ellipsoids drawn at the 30% probability level.

**Fig. 2.**
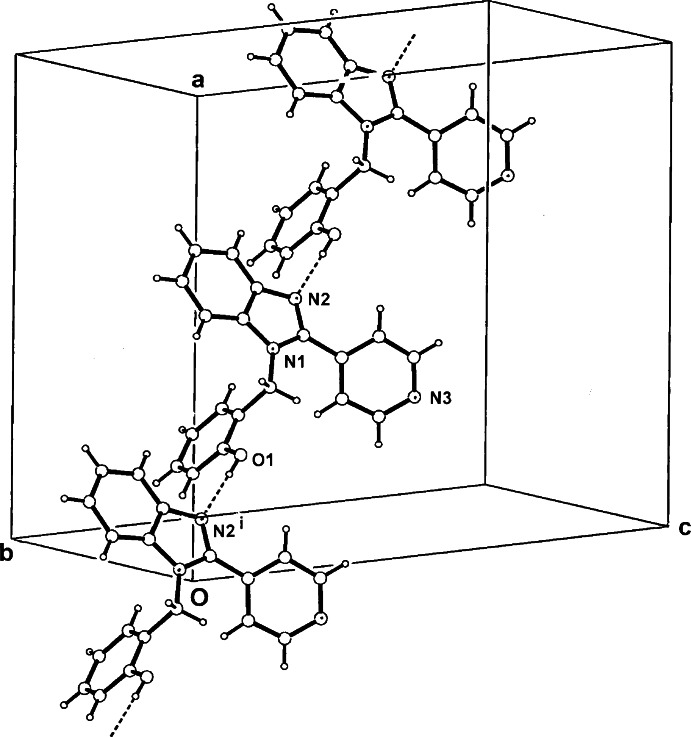
A perspective view of the packing of the title compound in the unit cell, showing intermolecular hydrogen bonds as dashed lines. For symmetry code (i), see Table 1.

## supplementary crystallographic information

### Comment

Supramolecular chemistry based on coordination compounds is a vast area of current research. Benzimidazole derivatives have attracted the attention of many synthetic chemists, due not only to their useful biological activities but also to their strong coordinating abilities as multidentate ligands (Kühler *et al.*, 2002; Carcanague *et al.*, 2002; Yang *et al.*, 2006; Li *et al.*, 2007). In our studies, we synthesized the substituted benzimidazole ligand 2-[2-(pyridin-4-yl)-1*H*-benzimidazol-1-ylmethyl]phenol, C_19_H_15_N_3_O, derived from the transformation of an unsymmetrical Schiff base and the structure is reported herein.

Within the ligand, the pyridine group is rigidly linked to the central C atom of the benzimidazole group, and the phenol ring is attached *via* a methylene group to an N-atom (Kitazume & Ishikawa, 1974). The dihedral angles between the pyridine and phenyl rings and the benzimidazole ring are 44.36 (7) and 75.67 (7)°, respectively. These deviations from planarity, in part, may be influenced by intermolecular phenolic O—H···N hydrogen bonds to benzimidazole N-atom acceptors (Table 1), giving one-dimensional chains extending along [110] (Fig. 2). In addition, the crystal structure is stabilized by weak face-to-face π–π stacking interactions involving the pyridine rings, with the shortest centroid to centroid distance between these planes of 3.9624 (10)Å]. This value is within the upper limit of the common range for π–π interactions (Janiak, 2000).

### Experimental

2-[2-(Pyridin-4-yl)-1*H*-benzimidazol-1-ylmethyl]phenol was synthesized according to a modification of a previously reported procedure (Fellah *et al.*, 2010). To a solution of *o*-phenylenediamine (2.16 g, 20 mmol) in ethanol (20 ml), 2-hydroxybenzaldehyde (1.22 g, 10 mmol) dissolved in ethanol (10 ml) was added dropwise. The mixture was stirred at room temperature for 8 h. A yellow precipitate formed and was isolated by filtration. The crude product,[(E)-2((2-aminophenylimino)methyl)], was then crystallized from ethanol and a solution of this product (2.12 g, 10 mmol) and pyridine-4-carbaldehyde (1.07 g, 10 mmol) in ethanol (50 ml) was heated for 10 h under reflux. The reaction mixture was cooled, and a white precipitate of the crude title compound formed and was filtered off (yield 71 wt%). This product was then recrystallized from an aqueous methanol solution (1:1 *v*/*v*). Colorless single block crystals of the title compound suitable for X-ray analysis were obtained.

### Refinement

The phenolic H atom was positioned and refined as a freely rotating O—H bond, with a fixed O—H bond length of 0.82 Å and with *U*_iso_(H) = 1.5*U*_eq_(O). Other H atoms were included in calculated positions and refined using a riding-model approximation, with C—H = 0.93 or 0.97 Å for aromatic and methylene H atoms, respectively, and with *U*_iso_(H) = 1.2*U*_eq_(C).

### Crystal data

**Table:**

C_19_H_15_N_3_O	*F*(000) = 1264
*M**_r_* = 301.34	*D*_x_ = 1.362 Mg m^−^^3^
Monoclinic, *C*2/*c*	Mo *K*α radiation, λ = 0.7107 Å
*a* = 16.1886 (7) Å	Cell parameters from 2313 reflections
*b* = 8.4863 (4) Å	θ = 3.1–28.4°
*c* = 21.4316 (10) Å	µ = 0.09 mm^−^^1^
β = 93.756 (4)°	*T* = 291 K
*V* = 2938.0 (2) Å^3^	Block, colourless
*Z* = 8	0.36 × 0.28 × 0.25 mm

### Data collection

**Table:**

Agilent SuperNova CCD diffractometer	3010 independent reflections
Radiation source: SuperNova (Mo) X-ray Source	2398 reflections with *I* > 2σ(*I*)
Mirror monochromator	*R*_int_ = 0.026
Detector resolution: 16.0733 pixels mm^-1^	θ_max_ = 26.4°, θ_min_ = 3.1°
ω scans	*h* = −19→20
Absorption correction: multi-scan (*CrysAlis PRO*; Agilent, 2012)	*k* = −10→8
*T*_min_ = 0.982, *T*_max_ = 1.000	*l* = −26→12
6104 measured reflections	

### Refinement

**Table:**

Refinement on *F*^2^	Primary atom site location: structure-invariant direct methods
Least-squares matrix: full	Secondary atom site location: difference Fourier map
*R*[*F*^2^ > 2σ(*F*^2^)] = 0.047	Hydrogen site location: inferred from neighbouring sites
*wR*(*F*^2^) = 0.119	H-atom parameters constrained
*S* = 1.06	*w* = 1/[σ^2^(*F*_o_^2^) + (0.0458*P*)^2^ + 1.9069*P*] where *P* = (*F*_o_^2^ + 2*F*_c_^2^)/3
3010 reflections	(Δ/σ)_max_ < 0.001
209 parameters	Δρ_max_ = 0.14 e Å^−^^3^
0 restraints	Δρ_min_ = −0.19 e Å^−^^3^

### Special details

**Table:**

Geometry. All e.s.d.'s (except the e.s.d. in the dihedral angle between two l.s. planes) are estimated using the full covariance matrix. The cell e.s.d.'s are taken into account individually in the estimation of e.s.d.'s in distances, angles and torsion angles; correlations between e.s.d.'s in cell parameters are only used when they are defined by crystal symmetry. An approximate (isotropic) treatment of cell e.s.d.'s is used for estimating e.s.d.'s involving l.s. planes.
Refinement. Refinement of *F*^2^ against ALL reflections. The weighted *R*-factor *wR* and goodness of fit *S* are based on *F*^2^, conventional *R*-factors *R* are based on *F*, with *F* set to zero for negative *F*^2^. The threshold expression of *F*^2^ > σ(*F*^2^) is used only for calculating *R*-factors(gt) *etc*. and is not relevant to the choice of reflections for refinement. *R*-factors based on *F*^2^ are statistically about twice as large as those based on *F*, and *R*- factors based on ALL data will be even larger.

### Fractional atomic coordinates and isotropic or equivalent isotropic displacement parameters (Å^2^)

**Table:**

	*x*	*y*	*z*	*U*_iso_*/*U*_eq_	
O1	0.14368 (7)	0.81061 (16)	0.41079 (6)	0.0423 (3)	
H1	0.0999	0.8588	0.4055	0.063*	
N1	0.38187 (8)	0.62034 (16)	0.40429 (6)	0.0283 (3)	
N2	0.49801 (8)	0.48060 (17)	0.39527 (7)	0.0336 (4)	
N3	0.32187 (10)	0.05156 (18)	0.48316 (8)	0.0428 (4)	
C1	0.43786 (10)	0.7203 (2)	0.37809 (8)	0.0295 (4)	
C2	0.43205 (11)	0.8769 (2)	0.35956 (9)	0.0389 (4)	
H2	0.3843	0.9358	0.3640	0.047*	
C3	0.50055 (12)	0.9406 (3)	0.33434 (10)	0.0478 (5)	
H3	0.4992	1.0456	0.3219	0.057*	
C4	0.57225 (12)	0.8523 (3)	0.32683 (10)	0.0475 (5)	
H4	0.6170	0.8993	0.3091	0.057*	
C5	0.57749 (11)	0.6972 (2)	0.34522 (9)	0.0417 (5)	
H5	0.6248	0.6381	0.3398	0.050*	
C6	0.50936 (10)	0.6314 (2)	0.37236 (8)	0.0318 (4)	
C7	0.42090 (9)	0.47799 (19)	0.41279 (8)	0.0281 (4)	
C8	0.38405 (10)	0.33423 (19)	0.43756 (7)	0.0283 (4)	
C9	0.30488 (10)	0.2814 (2)	0.41897 (8)	0.0325 (4)	
H9	0.2709	0.3400	0.3910	0.039*	
C10	0.27757 (12)	0.1415 (2)	0.44250 (9)	0.0381 (4)	
H10	0.2247	0.1075	0.4292	0.046*	
C11	0.39780 (12)	0.1031 (2)	0.50078 (9)	0.0433 (5)	
H11	0.4299	0.0425	0.5293	0.052*	
C12	0.43145 (11)	0.2401 (2)	0.47940 (8)	0.0378 (4)	
H12	0.4851	0.2695	0.4927	0.045*	
C13	0.29647 (9)	0.6653 (2)	0.41536 (8)	0.0289 (4)	
H13A	0.2968	0.7650	0.4375	0.035*	
H13B	0.2725	0.5864	0.4415	0.035*	
C14	0.24385 (10)	0.68048 (19)	0.35455 (8)	0.0274 (4)	
C15	0.16757 (10)	0.75871 (19)	0.35461 (8)	0.0296 (4)	
C16	0.12004 (11)	0.7808 (2)	0.29896 (9)	0.0365 (4)	
H16	0.0702	0.8353	0.2991	0.044*	
C17	0.14634 (11)	0.7224 (2)	0.24338 (9)	0.0413 (5)	
H17	0.1144	0.7380	0.2062	0.050*	
C18	0.21993 (12)	0.6410 (3)	0.24306 (9)	0.0437 (5)	
H18	0.2370	0.5990	0.2060	0.052*	
C19	0.26815 (10)	0.6220 (2)	0.29809 (8)	0.0355 (4)	
H19	0.3183	0.5687	0.2973	0.043*	

### Atomic displacement parameters (Å^2^)

**Table:**

	*U*^11^	*U*^22^	*U*^33^	*U*^12^	*U*^13^	*U*^23^
O1	0.0284 (7)	0.0537 (9)	0.0450 (7)	0.0191 (6)	0.0052 (5)	−0.0023 (6)
N1	0.0204 (6)	0.0291 (8)	0.0358 (8)	0.0049 (5)	0.0060 (6)	0.0042 (6)
N2	0.0233 (7)	0.0334 (8)	0.0449 (9)	0.0074 (6)	0.0073 (6)	0.0041 (7)
N3	0.0508 (10)	0.0338 (9)	0.0452 (9)	0.0011 (8)	0.0126 (8)	0.0048 (7)
C1	0.0226 (8)	0.0337 (9)	0.0324 (8)	0.0011 (7)	0.0037 (7)	0.0031 (7)
C2	0.0303 (9)	0.0353 (10)	0.0516 (11)	0.0054 (8)	0.0064 (8)	0.0087 (9)
C3	0.0403 (11)	0.0404 (11)	0.0630 (13)	−0.0016 (9)	0.0064 (10)	0.0189 (10)
C4	0.0314 (10)	0.0546 (13)	0.0571 (12)	−0.0072 (9)	0.0088 (9)	0.0173 (10)
C5	0.0225 (8)	0.0515 (12)	0.0519 (11)	0.0033 (8)	0.0086 (8)	0.0079 (10)
C6	0.0231 (8)	0.0346 (10)	0.0378 (9)	0.0022 (7)	0.0026 (7)	0.0050 (8)
C7	0.0222 (8)	0.0306 (9)	0.0313 (8)	0.0056 (7)	0.0017 (6)	0.0004 (7)
C8	0.0297 (8)	0.0273 (9)	0.0286 (8)	0.0056 (7)	0.0078 (7)	−0.0009 (7)
C9	0.0300 (9)	0.0347 (10)	0.0330 (9)	0.0033 (7)	0.0026 (7)	0.0020 (7)
C10	0.0384 (10)	0.0356 (10)	0.0411 (10)	−0.0040 (8)	0.0078 (8)	−0.0014 (8)
C11	0.0453 (11)	0.0409 (11)	0.0438 (11)	0.0093 (9)	0.0028 (9)	0.0131 (9)
C12	0.0332 (9)	0.0403 (11)	0.0394 (10)	0.0055 (8)	−0.0010 (8)	0.0048 (8)
C13	0.0217 (8)	0.0292 (9)	0.0366 (9)	0.0064 (7)	0.0082 (7)	0.0014 (7)
C14	0.0220 (7)	0.0244 (8)	0.0363 (9)	0.0016 (6)	0.0061 (7)	0.0025 (7)
C15	0.0226 (8)	0.0261 (9)	0.0405 (9)	0.0022 (7)	0.0052 (7)	0.0027 (7)
C16	0.0240 (8)	0.0363 (10)	0.0489 (11)	0.0016 (7)	0.0001 (8)	0.0074 (8)
C17	0.0342 (10)	0.0499 (12)	0.0391 (10)	−0.0072 (8)	−0.0037 (8)	0.0058 (9)
C18	0.0381 (10)	0.0574 (13)	0.0363 (10)	−0.0058 (9)	0.0077 (8)	−0.0041 (9)
C19	0.0257 (8)	0.0407 (11)	0.0410 (10)	0.0024 (8)	0.0079 (7)	−0.0019 (8)

### Geometric parameters (Å, º)

**Table:**

O1—H1	0.8200	C8—C12	1.393 (2)
O1—C15	1.362 (2)	C9—H9	0.9300
N1—C1	1.387 (2)	C9—C10	1.374 (2)
N1—C7	1.370 (2)	C10—H10	0.9300
N1—C13	1.4684 (19)	C11—H11	0.9300
N2—C6	1.387 (2)	C11—C12	1.376 (3)
N2—C7	1.327 (2)	C12—H12	0.9300
N3—C10	1.332 (2)	C13—H13A	0.9700
N3—C11	1.336 (3)	C13—H13B	0.9700
C1—C2	1.389 (2)	C13—C14	1.515 (2)
C1—C6	1.394 (2)	C14—C15	1.402 (2)
C2—H2	0.9300	C14—C19	1.388 (2)
C2—C3	1.376 (3)	C15—C16	1.389 (2)
C3—H3	0.9300	C16—H16	0.9300
C3—C4	1.399 (3)	C16—C17	1.383 (3)
C4—H4	0.9300	C17—H17	0.9300
C4—C5	1.375 (3)	C17—C18	1.378 (3)
C5—H5	0.9300	C18—H18	0.9300
C5—C6	1.397 (2)	C18—C19	1.380 (3)
C7—C8	1.472 (2)	C19—H19	0.9300
C8—C9	1.391 (2)		
			
C15—O1—H1	109.5	N3—C10—C9	124.25 (18)
C1—N1—C13	123.64 (13)	N3—C10—H10	117.9
C7—N1—C1	106.57 (13)	C9—C10—H10	117.9
C7—N1—C13	129.68 (14)	N3—C11—H11	118.0
C7—N2—C6	105.34 (14)	N3—C11—C12	124.03 (17)
C10—N3—C11	116.32 (16)	C12—C11—H11	118.0
N1—C1—C2	131.86 (15)	C8—C12—H12	120.5
N1—C1—C6	105.87 (14)	C11—C12—C8	119.07 (17)
C2—C1—C6	122.27 (15)	C11—C12—H12	120.5
C1—C2—H2	121.7	N1—C13—H13A	109.3
C3—C2—C1	116.55 (17)	N1—C13—H13B	109.3
C3—C2—H2	121.7	N1—C13—C14	111.43 (13)
C2—C3—H3	119.0	H13A—C13—H13B	108.0
C2—C3—C4	122.08 (18)	C14—C13—H13A	109.3
C4—C3—H3	119.0	C14—C13—H13B	109.3
C3—C4—H4	119.5	C15—C14—C13	119.01 (14)
C5—C4—C3	121.03 (17)	C19—C14—C13	122.95 (14)
C5—C4—H4	119.5	C19—C14—C15	118.03 (16)
C4—C5—H5	121.1	O1—C15—C14	117.08 (15)
C4—C5—C6	117.82 (17)	O1—C15—C16	122.82 (15)
C6—C5—H5	121.1	C16—C15—C14	120.10 (16)
N2—C6—C1	109.76 (14)	C15—C16—H16	119.8
N2—C6—C5	130.01 (16)	C17—C16—C15	120.43 (16)
C1—C6—C5	120.21 (16)	C17—C16—H16	119.8
N1—C7—C8	125.77 (14)	C16—C17—H17	120.0
N2—C7—N1	112.44 (15)	C18—C17—C16	119.96 (17)
N2—C7—C8	121.79 (14)	C18—C17—H17	120.0
C9—C8—C7	123.44 (15)	C17—C18—H18	120.2
C9—C8—C12	117.26 (16)	C17—C18—C19	119.66 (18)
C12—C8—C7	119.21 (15)	C19—C18—H18	120.2
C8—C9—H9	120.5	C14—C19—H19	119.1
C10—C9—C8	119.06 (16)	C18—C19—C14	121.77 (17)
C10—C9—H9	120.5	C18—C19—H19	119.1
			
O1—C15—C16—C17	−178.45 (17)	C7—N1—C1—C6	−0.32 (18)
N1—C1—C2—C3	−179.45 (18)	C7—N1—C13—C14	−104.93 (19)
N1—C1—C6—N2	−0.75 (19)	C7—N2—C6—C1	1.54 (19)
N1—C1—C6—C5	177.82 (16)	C7—N2—C6—C5	−176.85 (19)
N1—C7—C8—C9	44.3 (2)	C7—C8—C9—C10	176.76 (15)
N1—C7—C8—C12	−139.15 (18)	C7—C8—C12—C11	−177.78 (16)
N1—C13—C14—C15	−164.53 (14)	C8—C9—C10—N3	0.7 (3)
N1—C13—C14—C19	14.6 (2)	C9—C8—C12—C11	−1.0 (3)
N2—C7—C8—C9	−134.83 (18)	C10—N3—C11—C12	−0.2 (3)
N2—C7—C8—C12	41.7 (2)	C11—N3—C10—C9	−0.7 (3)
N3—C11—C12—C8	1.1 (3)	C12—C8—C9—C10	0.2 (2)
C1—N1—C7—N2	1.36 (19)	C13—N1—C1—C2	3.4 (3)
C1—N1—C7—C8	−177.87 (15)	C13—N1—C1—C6	−176.74 (14)
C1—N1—C13—C14	70.6 (2)	C13—N1—C7—N2	177.48 (15)
C1—C2—C3—C4	0.8 (3)	C13—N1—C7—C8	−1.7 (3)
C2—C1—C6—N2	179.12 (17)	C13—C14—C15—O1	−2.9 (2)
C2—C1—C6—C5	−2.3 (3)	C13—C14—C15—C16	176.89 (15)
C2—C3—C4—C5	−0.8 (3)	C13—C14—C19—C18	−178.34 (16)
C3—C4—C5—C6	−0.8 (3)	C14—C15—C16—C17	1.8 (3)
C4—C5—C6—N2	−179.47 (19)	C15—C14—C19—C18	0.8 (3)
C4—C5—C6—C1	2.3 (3)	C15—C16—C17—C18	0.3 (3)
C6—N2—C7—N1	−1.79 (19)	C16—C17—C18—C19	−1.8 (3)
C6—N2—C7—C8	177.48 (15)	C17—C18—C19—C14	1.2 (3)
C6—C1—C2—C3	0.7 (3)	C19—C14—C15—O1	177.92 (16)
C7—N1—C1—C2	179.83 (19)	C19—C14—C15—C16	−2.3 (2)

### Hydrogen-bond geometry (Å, º)

**Table:**

*D*—H···*A*	*D*—H	H···*A*	*D*···*A*	*D*—H···*A*
O1—H1···N2^i^	0.82	1.95	2.7659 (18)	177

Symmetry code: (i) *x*−1/2, *y*+1/2, *z*.
